# A Conserved EAR Motif Is Required for Avirulence and Stability of the *Ralstonia solanacearum* Effector PopP2 *In Planta*

**DOI:** 10.3389/fpls.2017.01330

**Published:** 2017-08-02

**Authors:** Cécile Segonzac, Toby E. Newman, Sera Choi, Jay Jayaraman, Du Seok Choi, Ga Young Jung, Heejung Cho, Young Kee Lee, Kee Hoon Sohn

**Affiliations:** ^1^Department of Life Sciences, Pohang University of Science and Technology Pohang, South Korea; ^2^Plant Science Department, Plant Genomics and Breeding Institute and Research Institute of Agriculture and Life Sciences, College of Agriculture and Life Sciences, Seoul National University Seoul, South Korea; ^3^Bioprotection Centre of Research Excellence, Institute of Agriculture and Environment, Massey University Palmerston North, New Zealand; ^4^National Institute of Agricultural Sciences, Rural Development Administration Wanju, South Korea; ^5^School of Interdisciplinary Bioscience and Bioengineering, Pohang University of Science and Technology Pohang, South Korea

**Keywords:** effector recognition, natural variation, repressor motif, transcriptional co-repressor, disease resistance

## Abstract

*Ralstonia solanacearum* is the causal agent of the devastating bacterial wilt disease in many high value *Solanaceae* crops. *R. solanacearum* secretes around 70 effectors into host cells in order to promote infection. Plants have, however, evolved specialized immune receptors that recognize corresponding effectors and confer qualitative disease resistance. In the model species *Arabidopsis thaliana*, the paired immune receptors RRS1 (resistance to *Ralstonia solanacearum* 1) and RPS4 (resistance to *Pseudomonas syringae* 4) cooperatively recognize the *R. solanacearum* effector PopP2 in the nuclei of infected cells. PopP2 is an acetyltransferase that binds to and acetylates the RRS1 WRKY DNA-binding domain resulting in reduced RRS1-DNA association thereby activating plant immunity. Here, we surveyed the naturally occurring variation in PopP2 sequence among the *R. solanacearum* strains isolated from diseased tomato and pepper fields across the Republic of Korea. Our analysis revealed high conservation of *popP2* sequence with only three polymorphic alleles present amongst 17 strains. Only one variation (a premature stop codon) caused the loss of RPS4/RRS1-dependent recognition in Arabidopsis. We also found that PopP2 harbors a putative eukaryotic transcriptional repressor motif (ethylene-responsive element binding factor-associated amphiphilic repression or EAR), which is known to be involved in the recruitment of transcriptional co-repressors. Remarkably, mutation of the EAR motif disabled PopP2 avirulence function as measured by the development of hypersensitive response, electrolyte leakage, defense marker gene expression and bacterial growth in Arabidopsis. This lack of recognition was partially but significantly reverted by the C-terminal addition of a synthetic EAR motif. We show that the EAR motif-dependent gain of avirulence correlated with the stability of the PopP2 protein. Furthermore, we demonstrated the requirement of the PopP2 EAR motif for PTI suppression. A yeast two-hybrid screen indicated that PopP2 does not interact with any well-known Arabidopsis transcriptional co-repressors. Overall, this study reveals high conservation of the PopP2 effector in Korean *R. solanacearum* strains isolated from commercially cultivated tomato and pepper genotypes. Importantly, our data also indicate that the PopP2 conserved repressor motif could contribute to the effector accumulation in plant cells.

## Introduction

The soil-borne pathogen *Ralstonia solanacearum* is the cause of devastating bacterial wilt in a wide range of host species including agronomically important *Solanaceae* species. Due to the wide genetic and host range diversity of strains, the concept of an *R. solanacearum* species complex (RSSC) is now generally accepted (Genin and Denny, 2012). The first RSSC sequenced strain, GMI1000, belongs to phylotype I isolated from tomato plants (Salanoubat et al., 2002). GMI1000 harbors around 70 predicted type 3 effectors (T3Es), also termed Rips (*Ralstonia* injected proteins), which are secreted into host cells to promote infection and enable bacterial growth (Mukaihara et al., 2010; Peeters et al., 2013). The *R. solanacearum* T3E repertoire is extensive when compared to other bacterial pathogens such as *Xanthomonas* spp. and *Pseudomonas syringae*, which both possess 30–40 T3Es (Alfano and Collmer, 2004; Büttner and Bonas, 2010). Moreover, further analysis of all sequenced *R. solanacearum* strains revealed that there is a large number of conserved core effectors (>30) (Genin and Denny, 2012). This suggests that the common ancestor already possessed a large arsenal of T3Es.

One of the primary roles for the T3Es is to dampen or suppress host defense responses. These responses are initially induced by the recognition of conserved microbial features termed pathogen/microbe-associated molecular patterns (PAMPs/MAMPs), such as flagellin, the building block of the bacterial flagellum or peptidoglycan from the bacterial envelope (Felix et al., 1999; Gust et al., 2007). Activation of the host pattern-recognition receptors (PRRs) by these molecules leads to pattern-triggered immunity (PTI), an efficient defense response impeding pathogen growth (Zipfel et al., 2004; Jones and Dangl, 2006). Consequently, numerous T3Es have been shown to target and inhibit components of the PTI signaling pathway, restoring susceptibility in the host plant (Macho and Zipfel, 2015). In turn, plants have evolved an intracellular set of immune receptors belonging to the nucleotide-binding leucine-rich repeat resistance (NLR) protein family that can detect corresponding T3Es and activate effector-triggered immunity (ETI) (Jones et al., 2016). The T3Es that activate ETI are termed avirulence proteins. ETI is often associated with a strong programmed cell death (PCD) of the infected cells, the hypersensitive response (HR), which participates in pathogen growth restriction (Dodds and Rathjen, 2010).

At least 7 of the ∼70 Rips trigger HR in *R. solanacearum* host species. The first identified avirulent Rip, AvrA, triggers HR in tobacco (*Nicotiana tabacum*) (Carney and Denny, 1990; Robertson et al., 2004). PopP1, Awr2, Awr5 and RipTPS also trigger HR in tobacco leaf cells (Poueymiro et al., 2009, 2014; Solé et al., 2012). Awr2 and Awr5 trigger HR in other *Nicotiana* species, while PopP1 also acts as an avirulence gene in a *Petunia* line (Lavie et al., 2002; Solé et al., 2012). In wild eggplant (*Solanum torvum*), the putative Zn-dependent protease, RipAX2 (formerly Rip36), induces a strong HR (Peeters et al., 2013; Nahar et al., 2014). Finally, the well-characterized acetyltransferase effector, PopP2, is one of two sequence-unrelated effectors that are recognized in the model plant species Arabidopsis by the paired NLRs RPS4 and RRS1-R (Deslandes et al., 2002, 2003; Narusaka et al., 2009). Besides PopP2 being the sole *R. solanacearum* effector recognized in Arabidopsis so far, the system allowing for its recognition is of particular interest due to the unusual structure of the RRS1-R receptor that harbors a WRKY-DNA binding domain (Deslandes et al., 2002). Multiple WRKY transcription factors (TFs) are involved notably in wound or defense response (Eulgem and Somssich, 2007). Of note, in the Arabidopsis ecotype Col-0, a shorter form of the RRS1 protein caused by a premature stop codon after the WRKY domain loses the ability to recognize PopP2 and is therefore denoted as RRS1-S (Deslandes et al., 2002). For the clarity of this report, we refer to *RRS1-R* (Ws-2), the allele conferring PopP2 recognition, as *RRS1*. Importantly, as a result of the conservation of immune signaling in plants, transgenic tomato expressing *RPS4* and *RRS1* from Arabidopsis are resistant to infection by *R. solanacearum* RS1002 strain that carries *popP2* (Narusaka et al., 2013). Considering the large plasticity of the *R. solanacearum* effector repertoire across the species complex and geographic regions, investigation into PopP2 contribution to virulence and the structural requirement for its recognition by the RPS4/RRS1 complex is essential to envisage the deployment of these *R* genes in crop species (Dangl et al., 2013; Peeters et al., 2013).

PopP2 belongs to the YopJ-like family of cysteine proteases, which share conserved catalytic triad residues [histidine (H), aspartate (D)/glutamate (E), cysteine (C)]. However, several YopJ-like effectors from mammals and plant pathogens can modify their host target by *trans*-acetylation rather than proteolytic activity (Ma and Ma, 2016). Indeed, PopP2 exhibits acetyltransferase activity, which is fully dependent on the catalytic cysteine residue, C321. Auto-acetylation of a lysine residue (K383) is required for the *trans*-acetylation activity of PopP2 and RPS4/RRS1-mediated recognition (Tasset et al., 2010). PopP2 co-localizes with RRS1 to the plant cell nucleus; however, the N-terminal 148 amino acids of PopP2 that include a putative nuclear localization signal (NLS) are dispensable for nuclear localization and avirulence (Deslandes et al., 2003; Sohn et al., 2014). Two recent studies brought evidence that PopP2 specifically targets the WRKY domain of RRS1. Acetylation of a key lysine residue in the RRS1 WRKY domain results in dissociation from the DNA and RPS4-dependent ETI. The RRS1 WRKY domain hence acts as a decoy to trap PopP2 activity, which may otherwise target WRKY TFs to disable plant defense (Le Roux et al., 2015; Sarris et al., 2015).

PopP2, though not belonging *per se* to the core-effector repertoire, contributes significantly to *R. solanacearum* virulence when present (Macho et al., 2010). This contribution to virulence could now be attributed to the ability of PopP2 to acetylate multiple host WRKY TFs probably resulting in their dissociation from DNA (Le Roux et al., 2015). WRKY TFs are integral for the regulation of plant innate immunity and are implicated in PTI, ETI and systemic acquired resistance (SAR) responses (Eulgem and Somssich, 2007; Rushton et al., 2010). PopP2-mediated WRKY TF acetylation has been shown to abrogate PAMP-triggered immunity (PTI) and contribute to *R. solanacearum* virulence (Le Roux et al., 2015). Thus, one can infer that the virulence function of the nuclear localized PopP2 is to manipulate host defense gene transcription via inhibition of WRKY TF DNA binding.

WRKY TFs play roles as both activators and repressors in plant immune signaling (Xu et al., 2006). Transcriptional repression is mainly achieved by chromatin modification at different levels (Berger, 2007). Transcriptional repressors can interact with co-repressors that recruit histone deacetylase for epigenetic silencing of gene expression (Long et al., 2006; Krogan et al., 2012). Direct interaction between repressor and co-repressor is mediated by the ethylene-responsive element binding factor-associated amphiphilic repression (EAR) motif (Song and Galbraith, 2006; Szemenyei et al., 2008). For instance, the co-repressor TOPLESS (TPL) is involved in the regulation of jasmonic acid (JA) signaling. Jasmonate ZIM-domain (JAZ) proteins function as transcriptional repressors of JA-regulated genes (Santner and Estelle, 2007). The majority of JAZ proteins directly bind to an adapter protein, Novel Interactor of JAZ (NINJA), which possesses an EAR motif and recruits TPL to epigenetically silence gene transcription via the histone deacetylase HDA19 (Pauwels et al., 2010; Pauwels and Goossens, 2011). TPL and TOPLESS-RELATED (TPRs) belong to the Groucho (Gro)/Tup1-like family of co-repressors encompassing 13 members in Arabidopsis (Liu and Karmarkar, 2008). Similar to TPL involvement in JA regulation of gene expression, TPRs play a role in the repression of negative regulators of defense during infection (Zhu et al., 2010). Although the recruitment of co-repressors for the repressor activity of some WRKY TFs has not been demonstrated yet, it is interesting to note that three members of the WRKY family contain an EAR motif (Kagale et al., 2010).

To gain further insights into the surveillance system that monitors virulence activity in the plant cell, we analyzed the natural sequence variation of PopP2 in *R. solanacearum* strains isolated from diseased tomato and pepper fields across Republic of Korea. We found that the sequence is highly conserved and identified that, among several, only one PopP2 allele lacks avirulence activity. This indicates that RPS4/RRS1-mediated recognition can tolerate multiple natural polymorphisms in PopP2. Furthermore, we identified a conserved EAR motif in PopP2, which we show to be required for *in planta* recognition, PTI suppression and protein accumulation. Besides providing valuable insight into the natural variation of PopP2 in virulent *R. solanacearum* strains, our study also unveils a novel mechanism by which a pathogenic effector could maintain its stability in the host cell.

## Results

### PopP2 Is Highly Conserved among Korean *R. solanacearum* Isolates and Harbors a Putative Transcriptional Repressor Motif

**FIGURE 1:**
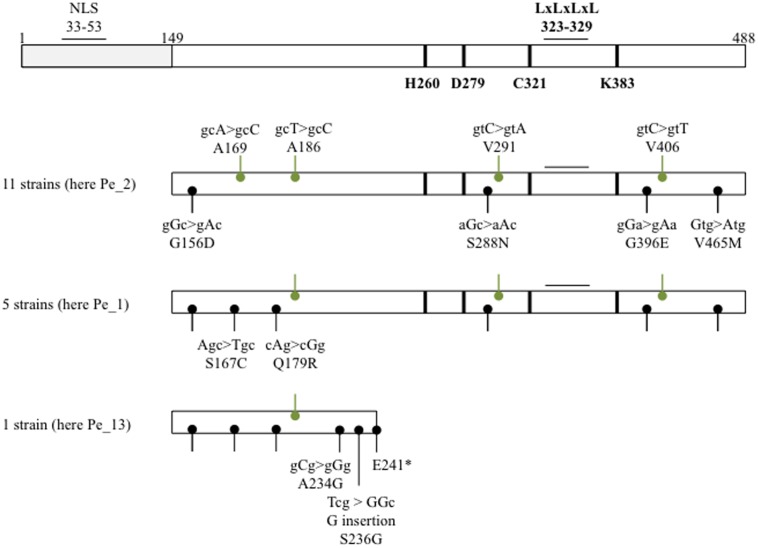
Natural variants and conserved residues of the PopP2 effector. A schematic of PopP2 sequence displaying natural variation and known or putative functional residues/motifs. GMI1000 PopP2 was used as the reference sequence. Labels in black show non-synonymous mutations and labels in green show synonymous mutations. See **Table 1** for further details about the strains. The asterisk indicates a stop codon.

In order to survey naturally occurring sequence variation in PopP2, we first selected 20 *R. solanacearum* strains isolated from commercially grown pepper or tomato plants showing wilting symptoms in the Republic of Korea, on the basis of their geographic location, the host plant they were collected from (Pepper, strains ‘Pe_’ and Tomato, strains ‘To_’) and the year of collection (**Table 1** and Supplementary Figure 1). Using gene-specific primers, we could amplify and confirm the presence of *popP2* in 17 of the 20 *R. solanacearum* strains (**Table 1**). The genomic sequence encoding the C-terminal region of PopP2 that is necessary and sufficient for avirulence in Arabidopsis, amino acids 149–488, was analyzed in the 17 *popP2*-harboring strains and compared to the GMI1000 reference (Salanoubat et al., 2002; Sohn et al., 2014). 11 strains (Pe_2, Pe_3, Pe_18, Pe_24, Pe_27, Pe_42, Pe_45, Pe_56, To_1, To_7, and To_42) harbored four SNPs resulting in the following amino acid residue changes: G156D, S288N, G396E, and V465M. These 11 strains also harbored four synonymous mutations at A169, A186, V291, and V406. Five other strains (Pe_1, Pe_26, Pe_28, Pe_40, and To_63) harbored the four aforementioned non-synonymous SNPs as well as two additional SNPs resulting in S167C and Q179R. These five isolates all harbored the same aforementioned synonymous SNPs except for the mutation at A169. Finally, the Pe_13 strain harbored G156D, S167C, and Q179R as well as the synonymous mutation at A186. In addition to this, Pe_13 harbored a SNP resulting in A234G and a single nucleotide insertion, which resulted in a premature stop codon, E241^∗^ (**Figure 1**). Therefore, our survey identified three novel polymorphic PopP2 variant groups, which we termed PopP2^Pe_2^ (four non-synonymous SNPs, present in 11 strains), PopP2^Pe_1^ (six non-synonymous SNPs, present in five strains) and PopP2^Pe_13^ (four non-synonymous SNPs and a frameshift insertion, present in only one of the selected strains) (**Figure 1**). The majority of strains analyzed here (11 of 17) harboring the “Pe_2” *popP2* allele were isolated from regions spanning the length of the Republic of Korea from Hwacheon in the north down to the southern coastal region, Haenam (Supplementary Figure 1). Among the five strains harboring the “Pe_1” allele, four were isolated from pepper fields in western regions; the other was isolated from tomato in eastern Bongwha. The Pe_13 strain carrying the truncated PopP2 variant was isolated from Imsil in the south–west (Supplementary Figure 1). The specific host cultivar genotypes are unknown. Thus, no obvious correlation between the host or the location of the isolated strains and the presence of a specific *popP2* allele could be revealed by this survey.

**Table 1:** Origin of the isolated *Ralstonia solanacearum* strains.

Name	Number	Host	Year of isolation	*popP2* presence
Pe_1	YKB3030	Pepper	2000	+
Pe_2	YKB3033	Pepper	2000	+
Pe_3	YKB3078	Pepper	2001	+
Pe_4	YKB4598	Pepper	2001	-
Pe_13	YKB5438	Pepper	2002	+
Pe_18	YKB5445	Pepper	2002	+
Pe_24	YKB5458	Pepper	2002	+
Pe_26	YKB5774	Pepper	2003	+
Pe_27	YKB5778	Pepper	2003	+
Pe_28	YKB5861	Pepper	1999	+
Pe_40	YKB6924	Pepper	2005	+
Pe_42	YKB6953	Pepper	2005	+
Pe_45	YKB7024	Pepper	2005	+
Pe_56	YKB7141	Pepper	2005	+
Pe_57	YKB7171	Pepper	2005	-
To_1	YKB9153	Tomato	2008	+
To_7	YKB9174	Tomato	2008	+
To_42	YKB9246	Tomato	2008	+
To_52	YKB9258	Tomato	2008	-
To_63	YKB9274	Tomato	2008	+

*List of the strains used in this study, including host and year of isolation. *popP2* presence as assessed by PCR amplification with specific primers is indicated by “+” and *popP2* absence is indicated by “-”*.

Nonetheless, closer analysis of the PopP2 coding sequence highlighted the presence of a putative LxLxL ethylene-responsive element binding factor-associated amphiphilic repression (EAR) motif; a motif involved in transcriptional repression via recruitment of transcriptional co-repressors (Hiratsu et al., 2003; Kagale and Rozwadowski, 2011). The reported motif comprises three leucine residues with amino acid spacers; however, PopP2 possesses an additional fourth leucine residue (LxLxLxL) at amino acids 323–329, which is almost adjacent to C321, one of the three conserved catalytic residues, H260, D279, and C321 (Tasset et al., 2010). Similarly to the catalytic residues, the putative EAR motif is fully conserved in all the sequenced PopP2 variants (**Figure 1**).

### Only One of the Newly Identified PopP2 Variants Loses Avirulence Function *In Planta*

**FIGURE 2:**
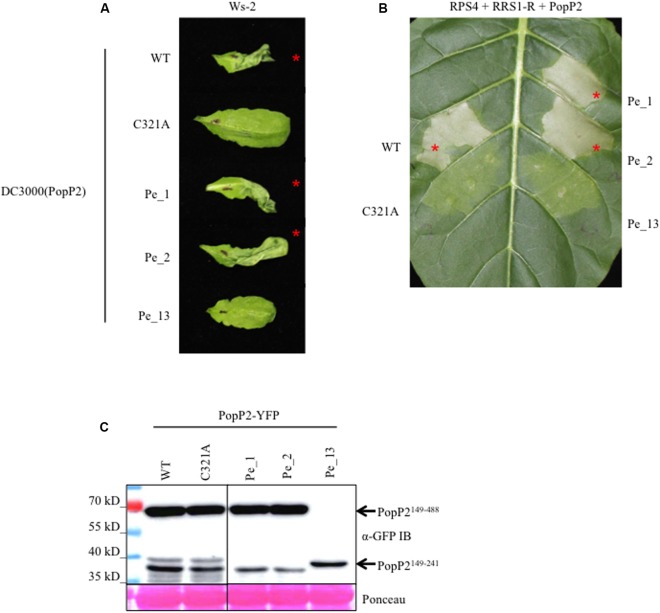
PopP2^Pe_1^ and PopP2^Pe_2^ are avirulent in Arabidopsis, PopP2^Pe_13^ is not. **(A)** PopP2 natural variants, PopP2^Pe_1^ and PopP2^Pe_2^, elicit an HR in resistant Arabidopsis accession Ws-2. The truncated natural variant, PopP2^Pe_13^ is not recognized. PopP2 variants were delivered by *Pseudomonas syringae* pv. *tomato (Pto)* DC3000 and photographs were taken 1 day post-infiltration (1 dpi). Red asterisks indicate HR. This experiment was conducted three times with similar results. **(B)** *Agrobacterium*-mediated co-expression of PopP2 variants with RPS4 and RRS1-R in tobacco leaf cells. Red asterisks indicate strong programmed cell death (PCD) at 3 dpi. This experiment was conducted three times with similar results. **(C)** The PopP2 natural variant proteins accumulate to a similar amount in *Nicotiana benthamiana* after agro-infiltration. Immuno-detection of PopP2 variants C-terminally fused to YFP epitope tag was conducted using anti-GFP antibodies. Ponceau S staining of total protein demonstrates equal loading of the samples.

To investigate the effect of natural polymorphism on the avirulence activity of PopP2, the three newly identified PopP2 variants lacking N-terminal region were translationally fused with AvrRps4 N-terminal domain (AvrRps4N) and delivered by the *P. syringae* pv. *tomato* (*Pto*) DC3000 type 3 secretion system into the resistant Ws-2 Arabidopsis accession, carrying functional RPS4 and RRS1-R, to assay for HR (Sohn et al., 2014). At 1 day post-infiltration (dpi) *Pto* DC3000-delivered AvrRps4N:PopP2^Pe_1^ and AvrRps4N:PopP2^Pe_2^ variants triggered a strong HR (**Figure 2A**). Conversely, the truncated AvrRps4N:PopP2^Pe_13^ could not trigger HR in Arabidopsis. This lack of avirulence activity was expected as the catalytic residues required for acetyltransferase activity and RPS4/RRS1-mediated recognition are absent in this variant due to a premature stop codon (Tasset et al., 2010; Sohn et al., 2014) (**Figure 1**). Similar recognition events were observed when PopP2 natural variants were co-expressed with RRS1 and RPS4 in tobacco leaf cells after *Agrobacterium*-mediated transient transformation (hereafter, agroinfiltration) (**Figure 2B**). Expression of all three protein variants was confirmed by immuno-detection in *Nicotiana benthamiana* leaf extracts (**Figure 2C**). Notably, none of the six identified SNPs present in the avirulent PopP2^Pe_1^ allele affected recognition by RPS4/RRS1 despite the close proximity of two polymorphisms to catalytic residues (S288N and G396E). This suggests that these polymorphisms do not impair PopP2 acetyltransferase activity and that RPS4/RRS1-mediated recognition can accommodate significant variation in the PopP2 sequence.

### The Conserved EAR Motif Is Required for PopP2 Avirulence Activity in Arabidopsis

To test if PopP2^149-488^ (hereafter referred to as PopP2) requires an LxLxL motif (hereafter; EAR motif) for avirulence function, we performed site-directed mutagenesis on the PopP2 LxLxLxL sequence to generate the LxAxAxL variant (hereafter, PopP2^LAAL^). Mutation of the two central leucine residues ensured that both LxLxL sequences were disrupted. Additionally, we fused a synthetic EAR motif, SRDX (LDLDLELRLGFA, derived from the SUPERMAN repressor domain) or a mutated version of the artificial EAR motif, srdx (FDFDFEFRLGFA) to the C-terminus of PopP2^C321A^ and PopP2^LAAL^ to test the specificity of EAR motif function (Hiratsu et al., 2003).

**FIGURE 3:**
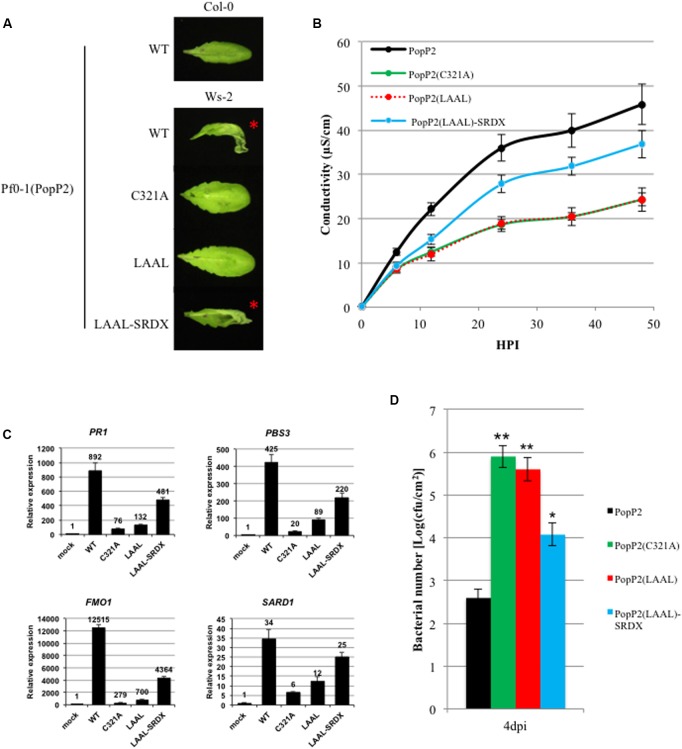
The EAR motif is required for PopP2 avirulence activity in Arabidopsis. **(A)** PopP2 EAR motif is required for HR elicitation in a resistant Arabidopsis accession (Ws-2). PopP2 variants were delivered by *Pseudomonas fluorescens* Pf0-1(T3S) and photographs were taken 1 day after infiltration. This experiment was conducted three times with similar results. **(B)** PopP2 EAR motif is required for ion leakage in Ws-2. Data are means ± SE (*n* = 6) of one representative experiment. This experiment was conducted three times with similar results. **(C)** PopP2 EAR motif is required for the upregulation of defense marker genes *PR1*, *PBS3*, *FMO1*, and *SARD1*. Values shown are the average of values obtained in three independent experiments ± SE. **(D)** PopP2 EAR motif is required for *Pto* DC3000 growth restriction. Data are shown as mean colony-forming units (cfu).cm^-2^ ± SE (*n* = 5). Asterisks indicate statistically significant differences from *Pto* DC3000(PopP2) growth (^∗^*P* < 0.01, ^∗∗^*P* < 0.001). This experiment was conducted three times with similar results.

To assay for HR in Arabidopsis, we used a modified *Pseudomonas fluorescens* strain, Pf0-1, which carries a functional type 3 secretion system [hereafter Pf0-1(T3S)] for delivery of the PopP2 variants (Thomas et al., 2009). As was previously reported, AvrRps4N:PopP2 triggered HR at 24 hours post-infection (hpi) in the resistant Ws-2 accession, but not in the susceptible Col-0 accession when delivered by Pf0-1(T3S) (Sohn et al., 2014). The catalytic cysteine mutant, PopP2^C321A^, was unable to trigger HR in Ws-2 due to the loss of acetyltransferase activity required for RPS4/RRS1-mediated recognition, and this was unaffected by fusion of C-terminal SRDX or srdx (Supplementary Figure 3) (Tasset et al., 2010; Sohn et al., 2014). Interestingly, the PopP2^LAAL^ variant with a disrupted EAR motif also failed to elicit HR in Ws-2. This suggests that PopP2 requires a functional EAR motif to trigger RPS4/RRS1-mediated HR. Indeed, fusion of the SRDX motif to the C-terminus of PopP2^LAAL^ partially restored the ability of PopP2 to trigger HR in Ws-2, suggesting that PopP2-triggered HR is dependent on a functional EAR motif. Fusion of the mutated artificial EAR motif, srdx, to PopP2^LAAL^ had no effect as expected (**Figure 3A**). PopP2 variants (WT, C321A, and LAAL) were delivered *in planta* as demonstrated by a secretion assay in *N. benthamiana* leaves (Supplementary Figure 5). In addition, we measured ion leakage to quantify the macroscopic HR symptoms and found that PopP2^LAAL^ induced ion leakage to the same extent as the negative control, PopP2^C321A^. In agreement with the HR data, PopP2^LAAL-SRDX^ induced more ion leakage than PopP2^LAAL^ to a level intermediate between PopP2^LAAL^ and PopP2^WT^ (**Figure 3B**).

It became apparent that the PopP2 EAR motif was required to trigger HR in Arabidopsis. However, HR does not always correlate with the defense response leading to immune transcriptional reprogramming (Yu et al., 1998; Cawly et al., 2005; Gassmann, 2005). Therefore, we also investigated the requirement of this motif for the induction of defense-related genes and disease resistance. To this end, we analyzed expression of multiple defense marker genes known to be upregulated by bacterial T3S-delivered PopP2: *PR1*, *FMO1*, *PBS3*, and *SARD1* (Sohn et al., 2014). The Arabidopsis accession Ws-2 was infiltrated with Pf0-1(T3S) carrying PopP2 variants and samples were taken 8 hpi for RNA extraction and qRT-PCR analysis. Consistent with the loss of HR induction by PopP2^LAAL^, the upregulation of all four defense genes was significantly impaired by mutation of the EAR motif. Additionally, fusion of the SRDX motif partially restored defense gene upregulation to a level more similar to PopP2^WT^ (**Figure 3C**). To further test the requirement of the EAR motif for PopP2 avirulence function, we assayed bacterial growth of *Pto* DC3000 carrying PopP2 variants in Arabidopsis (Sohn et al., 2014). As expected from our previous findings, *Pto* DC3000(PopP2^LAAL^) did not exhibit the growth restriction that PopP2^WT^ did, and grew to a number comparable to *Pto* DC3000 (PopP2^C321A^) (**Figure 3D**). Furthermore, *Pto* DC3000 (PopP2^LAAL-SRDX^) growth was partially restricted, corroborating our other evidence that PopP2 recognition *in planta* is dependent on a functional EAR motif. Overall, we have demonstrated that the PopP2 EAR motif is required for HR elicitation, upregulation of defense marker genes and bacterial growth restriction. The specificity of these effects of PopP2 EAR motif was further confirmed by the gain of avirulence observed with PopP2^LAAL^ fused to a C-terminal synthetic EAR motif.

### The Conserved EAR Motif Is Required for PopP2-Mediated PTI Suppression

Delivery of PopP2 but not PopP2^C321A^via *P. fluorescens* Pf0-1(T3S) has been demonstrated to inhibit PTI as indicated by cell death induction by subsequent *Pto* DC3000 infiltration (Crabill et al., 2010; Badel et al., 2013; Le Roux et al., 2015). This suggested that PopP2 acetyltransferase activity is required for virulence activity to suppress host PTI (Le Roux et al., 2015). Since discovering that the conserved EAR motif was required for avirulence, we sought to investigate its requirement for PopP2 virulence activity as measured by PTI suppression in *N. benthamiana* leaves. Infiltration of Pf0-1(T3S) carrying PopP2 variants alone [empty vector (EV), PopP2, PopP2^C321A^, PopP2^LAAL^, PopP2^LAAL-SRDX^, and PopP2^LAAL-SRDX^] induced no cell death while infiltration of *Pto* DC3000 carrying each aforementioned variant induced a cell death. As previously shown, infiltration of Pf0-1(T3S)(PopP2) followed by infiltration of *Pto* DC3000 resulted in a cell death response due to PopP2 PTI suppression; however, infiltration of Pf0-1(T3S)(EV) or Pf0-1(T3S) (PopP2^C321A^) followed by infiltration of *Pto* DC3000 resulted in no cell death induction due to *N. benthamiana* PTI-induced inhibition of *Pto* DC3000-induced cell death. Interestingly, we discovered that Pf0-1(T3S) (PopP2^LAAL^) infiltration into *N. benthamiana* leaves was also unable to suppress host PTI, as demonstrated by the lack of cell death induction by subsequent *Pto* DC3000 infiltration (Supplementary Figure 4). This suggests that the EAR motif is not only required for avirulence activity but also for PopP2 virulence function. Fusion of the artificial EAR motif, SRDX, partially restored the PTI suppression ability of PopP2, whereas fusion of the mutated version, srdx, had no effect (Supplementary Figure 4).

### PopP2 Does Not Interact with Known Arabidopsis Transcriptional Co-repressors in Yeast

The EAR motif is known to confer transcriptional repression activity via the recruitment of a co-repressor (Kagale and Rozwadowski, 2011). This led us to hypothesize that PopP2 recognition in Arabidopsis requires the recruitment of a transcriptional co-repressor. Therefore, we screened a library of transcriptional co-repressors for interaction with PopP2 using a LexA-based yeast-two-hybrid (Y2H) assay (Gyuris et al., 1993). The library we built comprises several members of the Groucho/Tup1-like family of co-repressors with known LisH domains and WD repeats (Supplementary Table 1) (Liu and Karmarkar, 2008). LEUNIG (LUG) and the closely related LEUNIG_HOMOLOG (LUH) as well as TPL and its close homologs TPR1–4 are the best characterized of the Gro/Tup1-like co-repressors. SEUSS (SEU) and its close homologs, SEUSS-LIKE (SLK1–2), can interact with LUG or LUH to form a functional repressor complex (Franks et al., 2002; Sitaraman et al., 2008; Grigorova et al., 2011; Shrestha et al., 2014). Finally, high expression of osmotically responsive genes 15 (HOS15) and SIN3-associated polypeptide of 18 kDa (SAP18) were included, as they are known to mediate transcriptional repression in Arabidopsis via chromatin modification (Song and Galbraith, 2006; Hill et al., 2008; Zhu et al., 2008).

Protein fusions were assayed for interaction in yeast with *LEU2* and *lacZ* reporter genes under the control of upstream LexA operators. Yeast cells expressing empty vector controls with fusion proteins were assayed for growth and the development of blue color on the induction medium [-His(H)/-Trp(T)/-Ura(U)/-Leu(L)] + X-Gal, to test for auto-activity. TPL, TPR4, and SAP18 in the pB42-AD vector alone activated *LEU2* but not *lacZ* allowing growth on medium lacking leucine; all other yeast cells did not activate reporter genes (Supplementary Figure 2). As expected, LUG interaction with SLK2, used as a positive control, showed strong interaction (Stahle et al., 2009). However, none of the yeast cells co-expressing PopP2-DBD and a transcriptional co-repressor-AD fusion protein showed clear protein-protein interaction in yeast cells (Supplementary Figure 2).

### The EAR Motif Is Required for PopP2 Stability *In Planta*

**FIGURE 4:**
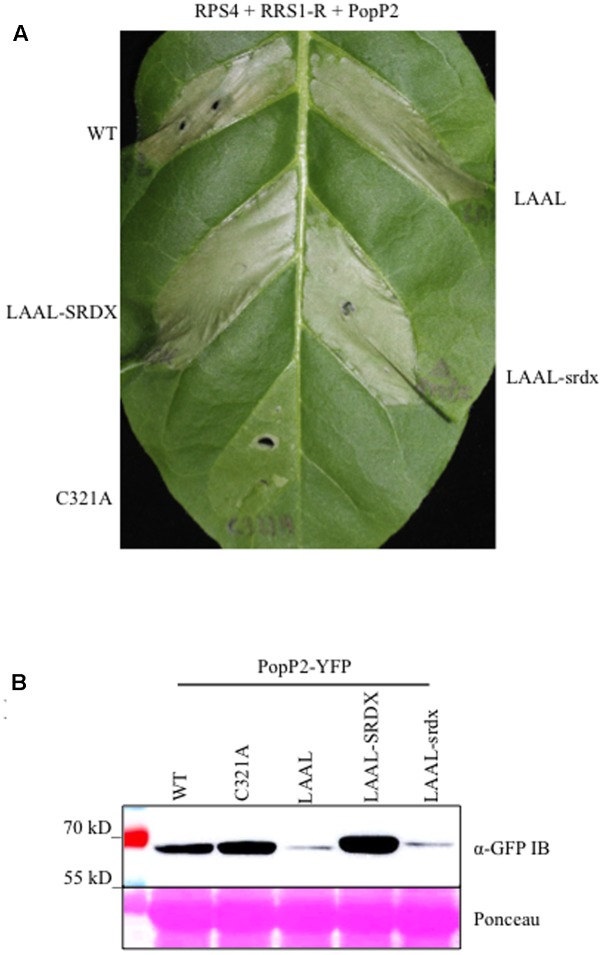
The EAR motif is required for PopP2 stability *in planta*. **(A)** Agro-mediated co-expression of the PopP2 variants with RPS4 and RRS1-R in tobacco. PopP2^LAAL^ triggers a cell death response when co-expressed with RPS4 and RRS1-R independent of SRDX fusion. This experiment was conducted 3 times with similar results. **(B)** Mutation of the PopP2 EAR motif affects PopP2 protein accumulation after transient expression following agro-infiltration in *N. benthamiana*. The wild-type level of protein accumulation could be restored by fusion of a functional synthetic EAR motif (SRDX), but not by the mutated version (srdx). Immuno-detection of PopP2 variants C-terminally fused to YFP tag was conducted using anti-GFP antibodies. Ponceau S staining of total protein demonstrates equal loading of the samples.

We have assayed the HR activation in response to PopP2 variants in the native Arabidopsis system; we also used a heterologous tobacco overexpression system to assay for RPS4/RRS1 mediated recognition of PopP2 variants (**Figure 4A**). Agroinfiltration of PopP2^WT^, RPS4, and RRS1 resulted in a robust PCD response at 3 dpi in tobacco. Conversely, co-expression of the inactive PopP2^C321A^ mutant with RPS4 and RRS1 resulted in significantly reduced PCD (Sohn et al., 2014). Intriguingly, the PopP2^LAAL^, PopP2^LAAL^-SRDX, and PopP2^LAAL^-srdx variants all elicited a strong PCD response when co-expressed with RPS4 and RRS1 (**Figure 4A**). Thus, loss of PopP2 avirulence activity as a result of EAR motif disruption in Arabidopsis could not be reconstituted in the tobacco overexpression system.

To confirm the expression of PopP2 variants, sequences were fused with a C-terminal YFP epitope tag and transiently expressed in *N. benthamiana*. Total proteins were extracted and subjected to immuno-detection with an anti-GFP antibody. PopP2^WT^ and PopP2^C321A^ accumulated to similar amounts. Surprisingly, PopP2^LAAL^ protein accumulation was significantly lower as indicated by the low intensity band on the blot (**Figure 4B**). Furthermore, fusion of the synthetic EAR motif restored the protein level to the WT level while fusion of the non-functional srdx motif, had no effect on protein accumulation. This evidence suggests that the stability of PopP2 inside plant cells is dependent on an EAR motif (**Figure 4B**).

## Discussion

The *R. solanacearum* acetyltransferase effector, PopP2, is known to activate RPS4/RRS1-mediated resistance in certain Arabidopsis accessions (Deslandes et al., 2003; Sohn et al., 2014). PopP2 acetyltransferase activity is required for auto-acetylation and *trans*-acetylation of key lysine residues in the RRS1 WRKY domain and activation of the RPS4/RRS1 immune complex (Tasset et al., 2010; Le Roux et al., 2015; Sarris et al., 2015). Here, our results indicate that while the RPS4/RRS1 immune complex can recognize the naturally occurring alleles of PopP2 in Korean *R. solanacearum* isolates, a conserved EAR motif is necessary for its avirulence activity by regulating the protein stability in the plant cell.

### PopP2 Natural Variation in Virulent *Ralstonia solanacearum* Strains

We surveyed the natural variation of PopP2 sequence in *R. solanacearum* strains isolated from diseased tomato and pepper fields at different geographic locations across the Republic of Korea. Gene-specific sequencing and subsequent analysis revealed that *popP2* is highly conserved across South Korean isolates, as we identified only three polymorphic alleles among the 17 *popP2*-harboring strains. In fact, all but two of the polymorphisms are conservative; they result in a change to an amino acid with similar properties. However, G156D and G396E result in a change from a single hydrogen radical to a negatively charged side chain. We have shown that, despite these polymorphisms, two of the natural variants, PopP2^Pe_1^ and PopP2^Pe_2^, retained avirulence activity in Arabidopsis Ws-2. The significantly truncated variant, PopP2^Pe_13^, did not trigger HR. This is consistent with the previous report showing that the catalytic triad is required for PopP2 acetyltransferase activity (Tasset et al., 2010).

It must be considered that the *R. solanacearum* strains from which the *popP2* alleles were isolated were highly virulent on pepper or tomato plants. Genome sequence analysis does not reveal any strong homology of *Solanaceae* disease resistance (*R*) genes with *RPS4* and *RRS1* (Consortium, 2012; Kim et al., 2014). However, the existence of PopP2^Pe_13^ (a truncated variant that lacks avirulence) suggests that there might be a selective pressure in natural host plants of *R. solanacearum*. This hypothesis is further supported by the three *R. solanacearum* strains that were shown to lack *popP2* in our study. In this regard, it would be interesting to survey the presence/absence and sequence polymorphism of *popP2* in *R. solanacearum* strains from other host plants or geographic regions. Furthermore, in addition to RPS4/RRS1, it is plausible that PopP2 may be recognized by other R protein(s) and that natural variants found in our study may show altered avirulence activity.

### EAR Motif-Dependent Protein Stability Control

Our study illustrates the first example of a plant pathogenic effector that is dependent on an EAR motif for avirulence activity. Of note, the *Xanthomonas campestris* pv. *vesicatoria* (*Xcv*) effector XopD possesses two EAR motifs [sequence (L/F)DLN(L/F)(X)P] that are both required for virulence. XopD represses defense gene transcription via the two EAR motifs to enable *Xcv* growth in tomato (Kim et al., 2008). Likewise, we demonstrated that the PopP2 EAR motif is required for PTI suppression, which may contribute to *R. solanacearum* virulence. In addition, we showed that disruption of the LxLxLxL amino acid sequence rendered PopP2 unstable, but could be stabilized by addition of the synthetic EAR motif SRDX at the C-terminus. Therefore, PopP2 stability appears to depend specifically on the presence of the LxLxLxL sequence. In the tobacco heterologous system, despite being clearly reduced, accumulation of PopP2^LAAL^ might nonetheless reach a threshold required for detection by the over-expressed RPS4 and RRS1 receptors. Conversely, in the native Arabidopsis system, we can infer that PopP2 EAR motif could play an important role in protein stability, allowing its accumulation above the necessary amount to trigger RRS1 activation.

A novel mechanism of controlling protein stability via an EAR motif has recently been unveiled. Proteasomal and non-proteasomal degradation of the ZINC FINGER OF ARABIDOPSIS THALIANA12 (ZAT12) TF is controlled by an LxLxL EAR motif (Le et al., 2016). Similarly to the reduced accumulation of PopP2^LAAL^, the abundance of the ZAT12 variant carrying a mutation in the EAR motif was lower than the wild-type. Le et al. (2016) hypothesized that the ZAT12 EAR motif is involved in mediating interactions with different partners; at least one of which is H_2_O_2_ responsive and another that is a factor of proteasomal degradation targeting, such as an E3-ubiquitin ligase. Conversely, an earlier study investigating a poplar (*Populus* spp.) ortholog of ZAT12, *Pti* Cys2/His2 zinc-finger protein 1 (*Pti*ZFP1), reported that the *Pti*ZFP1 EAR motif promotes its degradation by the 26S proteasome through MAPK binding (Hamel et al., 2011). This is in contrast to the ZAT12 EAR motif-mediated protein stability model, but it provides additional clues to investigate the mechanism regulating PopP2 accumulation *in planta*.

PopP2 contributes to *R. solanacearum* virulence in tomato, eggplant, bean, and Arabidopsis (Macho et al., 2010; Le Roux et al., 2015). It is conceivable that successful *R. solanacearum* strains have acquired PopP2 variants carrying an EAR motif to stabilize the secreted effector by circumventing *in planta* degradation. This mechanism could have been selected to enhance the virulence of *R. solanacearum* on host plants. Indeed, we have shown that PopP2 stability is dependent on a functional EAR motif and that this is associated with both PopP2-mediated PTI suppression and host recognition ability.

### Possible Mechanisms of PopP2 EAR Motif Function

The requirement of PopP2 for an EAR motif to trigger an avirulence response coupled with the numerous reports of EAR motif-mediated recruitment of co-repressors led us to generate yeast two-hybrid constructs of known Arabidopsis transcriptional co-repressors to screen for interaction with PopP2 in yeast cells. The Groucho (Gro)/Tup1-like family of co-repressors make up the largest and best characterized family of co-repressors in Arabidopsis with at least 13 members, including LUG and LUH, TPL and TPRs and HOS15 (Liu and Karmarkar, 2008). LUG and LUH are partially redundant transcriptional co-repressors and together with SEU/SLKs are involved in embryo and floral development and abiotic stress responses (Sitaraman et al., 2008; Shrestha et al., 2014). Similarly, HOS15 and SAP18 have not been implicated in plant defense so far, but mediate gene repression in response to cold or salt stress (Song and Galbraith, 2006; Hill et al., 2008; Zhu et al., 2008). On the other hand, the recruitment of the functionally redundant TPL and TPR1–4 by EAR motif-containing proteins in defense signaling is well-known. TPL is involved in the regulation of JA signaling for disease resistance to necrotrophic pathogens and stomatal defense (Pauwels et al., 2010; Pauwels and Goossens, 2011). Similarly, TPR1 and other TPRs associate with the *R* gene *SNC1* to participate in transcriptional repression of negative regulators of defense (Zhu et al., 2010).

Our yeast-two-hybrid (Y2H) screen data indicate that PopP2 does not interact directly with any of the tested co-repressors in our experimental conditions. Further confirmation of this result could be obtained by testing *in planta* interaction between PopP2 and transcriptional co-repressors in the future. Nonetheless, based on our findings, we hypothesize that *in planta* PopP2 stability may be dependent on EAR motif-mediated recruitment of an as yet untested co-repressor or another host component. Identification of the host factor(s) controlling PopP2 stability shall bring new insights into the systems used to monitor pathogen virulence in the plant cell.

## Materials and Methods

### Plant Materials and Growth Conditions

Arabidopsis accessions Col-0 and Ws-2 were grown in short day conditions (11 h light/13 h dark) at 22°C. *N. benthamiana* and *N. tabacum* W38 plants were grown in long day conditions (14 h light/10 h dark) at 25°C.

### Plasmid Constructions

For *Pseudomonas*-mediated delivery, *R. solanacearum* GMI1000 PopP2^149-488^ variants (WT, C321A, LAAL, LAAL-SRDX, and LAAL-srdx) were cloned in the pEDV6 gateway destination vector, which is described in detail in Sohn et al. (2014). The SRDX synthetic EAR motif, LDLDLELRLGFA, is described in Hiratsu et al. (2003). The mutated SRDX, termed srdx, encodes the peptide sequence FDFDFEFRLGFA. SRDX and srdx were fused to the *popP2* C-terminus with the cloning primers.

The natural polymorphic *popP2* variants (Pe_1, Pe_2, and Pe_13) were amplified from *R. solanacearum* strains isolated from pepper in Korean fields using gene-specific primers. These were cloned into a modified Golden Gate-compatible pBBR1MCS-5 broad host range vector containing *avrRps4* promoter (128 bp) and N-terminus (408 bp), as was used for the pEDV vectors (Sohn et al., 2007). *popP2* variants were fused to a C-terminal 6xHA tag.

Golden Gate-compatible pLexA-DBD and pB42-AD vectors were used for the Y2H assay and coding sequences were fused to C-terminal 3xFLAG and 6xHA, respectively. *popP2*(149–488) was amplified from a previously reported plasmid, pCR8:*popP2* (Sohn et al., 2014). Transcriptional co-repressors were amplified from Arabidopsis cDNA (accession Col-0).

The binary vector pICH86988 (provided by Sylvestre Marillonnet) was used for *Agrobacterium*-mediated delivery into *N. tabacum* and *N. benthamiana*. This is a Golden Gate-compatible vector in which *popP2* variants, *RPS4* (Arabidopsis accession No-0) and *RRS1-R* (Arabidopsis accession Ws-2) were assembled in fusion with C-terminal YFP, 3xHA and 3xFLAG, respectively.

For assembly into all Golden Gate-compatible vectors, genes were PCR-amplified using gene-specific primers, which also introduced *Bsa*I recognition sequences and specific 4 bp overhangs flanking the sequences. These were cloned into the pICH41021 shuttle vector (modified pUC19 with a mutated *Bsa*I recognition sequence). Golden Gate assembly of the resulting pICH41021 constructs into Golden Gate-compatible destination vectors was carried out to generate final constructs (Engler et al., 2008).

### Bacterial Strains, Culture Conditions, and Manipulations

*Escherichia coli* DH5α was used to maintain and replicate plasmids, as well as for bacterial conjugation. Modified *P. fluorescens* Pf0-1(T3S) was used for HR assays and ion leakage assays (Thomas et al., 2009). *P. syringae* pv. *tomato* (*Pto*) DC3000 was used for HR assays and *in planta* bacterial growth assays. To transform Pf0-1(T3S) and *Pto* DC3000 with the appropriate construct, we used triparental mating with *E. coli* HB101 (pRK2013) as a helper strain (Sohn et al., 2007) or *Pseudomonas* electroporation (Choi et al., 2006). *Agrobacterium tumefaciens* strain AGL1 was transformed by electroporation with appropriate binary constructs for the transient expression assays.

### Plant Pathology Experiments

For HR assays, bacteria were grown on King’s B (KB) agar containing appropriate antibiotics and harvested in 10 mM MgCl_2_. The final concentration of Pf0-1(T3S) suspensions was adjusted to OD_600_ = 0.2; the final concentration of *Pto* DC3000 suspensions was adjusted to OD_600_ = 0.1. Leaves of 5-week old Arabidopsis plants were hand-infiltrated on the abaxial surface using a blunt-end syringe, and macroscopic symptoms were observed and photographed at 20–24 hours post-infiltration (hpi). For ion leakage assays with Pf0-1(T3S), leaf disks were sampled at 0.5 hpi, washed in water for 30 min (with gentle shaking at room temperature) and transferred to fresh water (0 hpi sample). Ion leakage measurements were taken at 6, 12, 24, 36, and 48 hpi using a conductivity meter (Horiba B-173). For *in planta* bacterial growth assays, *Pto* DC3000 strains were grown and harvested as for HR assays; however, bacterial suspensions were adjusted to OD_600_ = 0.001. Leaves of 5-week old Arabidopsis plants were hand-infiltrated on the abaxial surface using a blunt-end syringe, and sampled at 4 days post-infiltration (dpi). Samples were ground in 10 mM MgCl_2_, serially diluted and spotted on KB agar containing appropriate antibiotics. These were incubated at 28°C for 2 days prior to counting colonies in order to calculate the number of colony forming units (cfu)/cm^2^ of infected leaf.

### Yeast-Two-Hybrid (Y2H) Assays

The *Saccharomyces cerevisiae* strains used for the Y2H assays were EGY48 Mat(α) and RFY206 Mat(a). RFY206 carries the pSH18–34 vector, which encodes the lacZ reporter gene under the control of 8 upstream LexA operators. Additionally, pSH18–34 encodes the URA3 selectable marker, allowing growth on media lacking uracil. EGY48 and RFY206(pSH18–34) were transformed with pB42-AD and pLexA-DBD constructs, respectively, using the ‘Frozen-EZ Yeast Transformation II Kit’ according to the manufacturer’s recommendations (Zymo Research). pB42-AD encodes the TRP1 selectable marker, which allows yeast growth on media lacking tryptophan (Trp), pLexA encodes the HIS3 selectable marker, allowing growth on media lacking histidine (His). After transformation of yeast with the appropriate constructs, mating and interaction assays were performed as described in the Yeast Protocols Handbook (Clontech).

### *Agrobacterium*-Mediated Transient Transformation of *Nicotiana benthamiana* and *Nicotiana tabacum*

*Agrobacterium tumefaciens* AGL1 carrying the binary constructs were grown in liquid L-medium supplemented with the appropriate antibiotics for 24 h. Cells were harvested by centrifugation and re-suspended in infiltration medium (10 mM MgCl_2_ and 10 mM MES-KOH pH 5.6). Suspensions were then adjusted to OD_600_ = 0.1. Bacterial suspensions were mixed in a 1:1 ratio and infiltrated into the abaxial surface of 5-week old *N. benthamiana* or *N. tabacum* leaves using a blunt-end syringe. Cell death was observed and photographed at 2–3 dpi.

### Protein Extraction and Immunoblotting

Plant protein samples were prepared from *N. benthamiana* 48 h after *Agrobacterium*-mediated transformation. One full infiltrated leaf was ground in liquid nitrogen and total proteins were extracted in GTEN buffer (10% glycerol, 150 mM Tris-HCl pH 7.5, 1 mM EDTA, 150 mM NaCl) supplemented with 5 mM DTT, plant protease inhibitor tablet (1 tablet/50 ml extraction solution) (Roche) and 0.2% (vol/vol) Nonidet P-40. Lysates were centrifuged for 15 min at 5000 rpm at 4°C. The supernatants were filtered through miracloth mesh (Millipore) and used as input samples. Proteins were separated by SDS-PAGE and immunoblotted using anti-GFP-HRP conjugated antibodies (Santa Cruz). Proteins were detected with a mix of SuperSignal West Pico and SuperSignal West Femto chemiluminescent substrates (Pierce). Membranes were stained with Ponceau S to visualize protein loading.

## Author Contributions

CS, TN, and KS conceived and designed the study. CS, TN, SC, JJ, DC, GJ, and KS carried out the experiments. HC and YL provided the *R. solanacearum* isolates and related information. CS, TN, and KS analyzed and interpreted the data. CS, TN, and KS prepared the manuscript.

## Conflict of Interest Statement

The authors declare that the research was conducted in the absence of any commercial or financial relationships that could be construed as a potential conflict of interest. The reviewer LD and handling Editor declared their shared affiliation, and the handling Editor states that the process met the standards of a fair and objective review.
