# Supplementary material for: A Conserved EAR Motif Is Required for Avirulence and Stability of the Ralstonia solanacearum Effector PopP2 In Planta
Source: Front Plant Sci. 2017 Aug 2;8:1330. doi: 10.3389/fpls.2017.01330 (PMC5539180; doi:10.3389/fpls.2017.01330)
Supplement: Supplementary file 1 [file Presentation_1.pdf]

Supplementary Material

A conserved EAR motif is required for avirulence and stability of the *Ralstonia solanacearum* effector PopP2 *in planta*

Cécile Segonzac[†*], Toby E. Newman[†], Sera Choi, Jay Jayaraman, Du Seok Choi, Gayoung Jung, Heejung Cho, Young Kee Lee, Kee Hoon Sohn[*]

[†]These authors have contributed equally to this work

*Correspondence: khsohn@postech.ac.kr, csegonzac@snu.ac.kr

The Supplementary Material for this article includes 5 figures, 1 table and references.

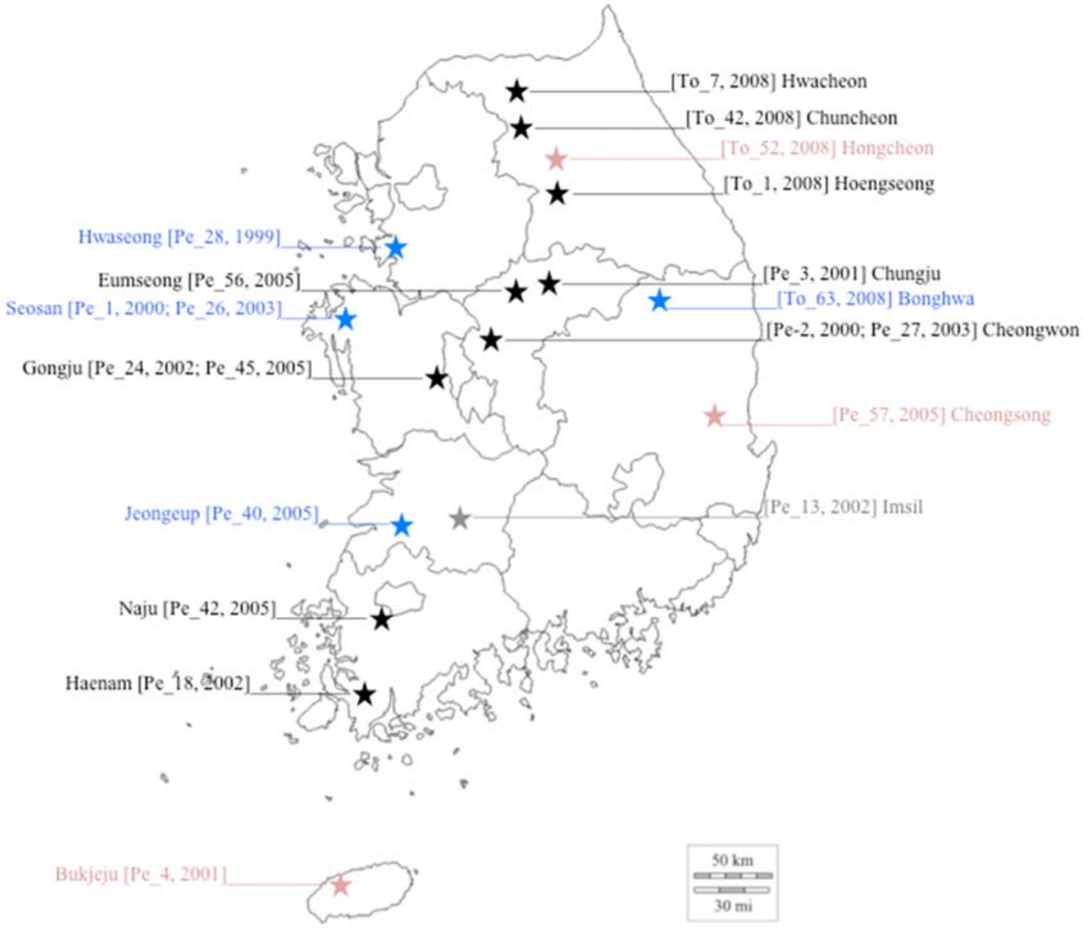

Supplementary Figure 1. Regional map of the Republic of Korea with location of strain isolation. Strains labelled in pink did not harbor *popP2* (Pe_4, Pe_57, To_52). Strains labelled in blue harbored the “Pe_1” allele (Pe_1, Pe_26, Pe_28, Pe_40 and To_63). Strains labelled in black harbored the “Pe_2” allele (Pe_2, Pe_3, Pe_18, Pe_24, Pe_27, Pe_45, Pe_56, To_1, To_7 and To_42). Pe_13 is labelled in grey.

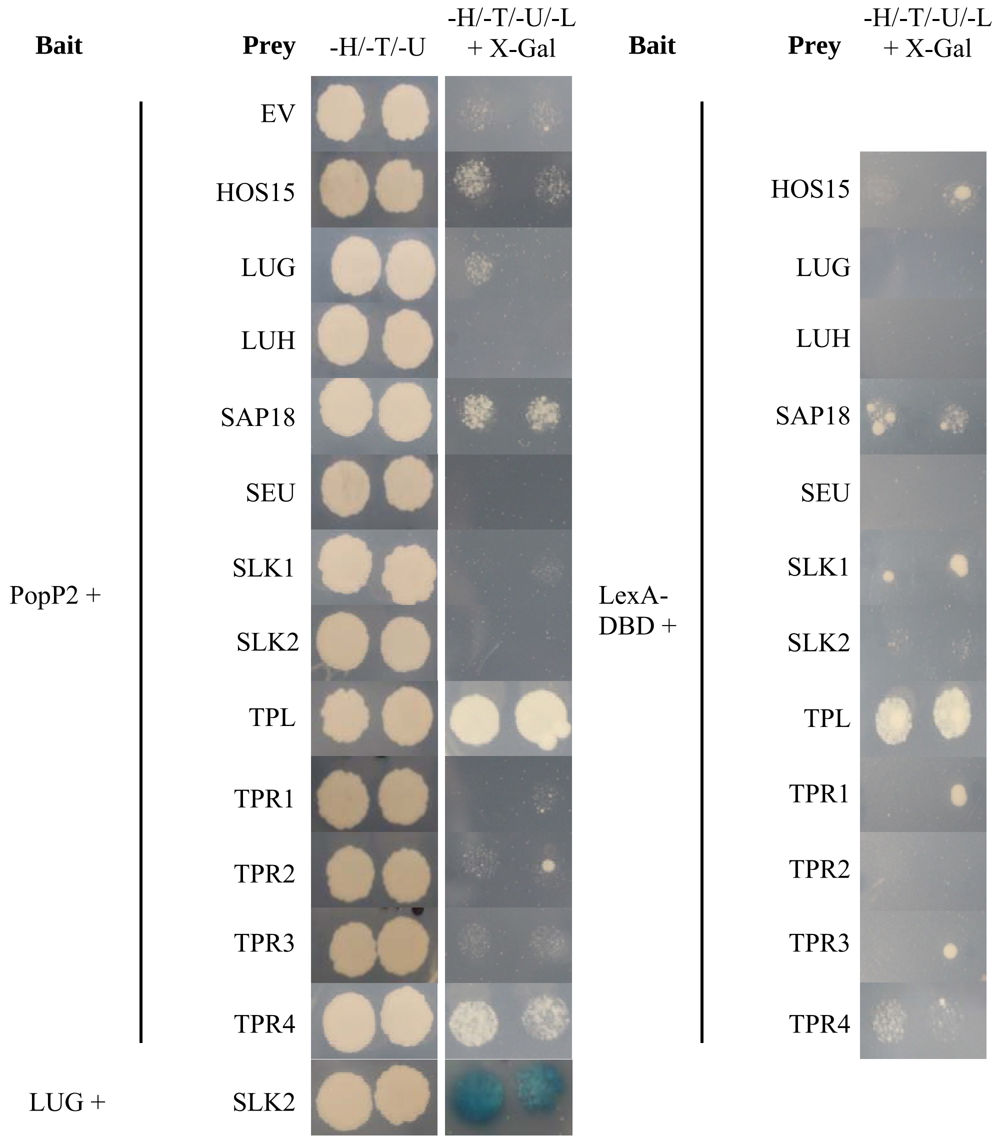

Supplementary Figure 2. PopP2 does not interact with known transcriptional co-repressors in yeast. Growth of yeast cells expressing LexA-DNA-binding domain (DBD) fusion (bait) and B42-activation domain (AD) fusion (prey) as indicated. Yeast cells were grown on non-selective media lacking histidine, tryptophan and uracil (-H/-T/-U) or selective media also lacking leucine (-H/-T/-U/-L) and supplemented with X-Gal. TPL, TPR4 and SAP18 B42-AD fusions are weakly auto-active as indicated by the growth of yeast cells expressing LexA-DBD alone and B42-AD protein fusions. This experiment was conducted twice with similar results.

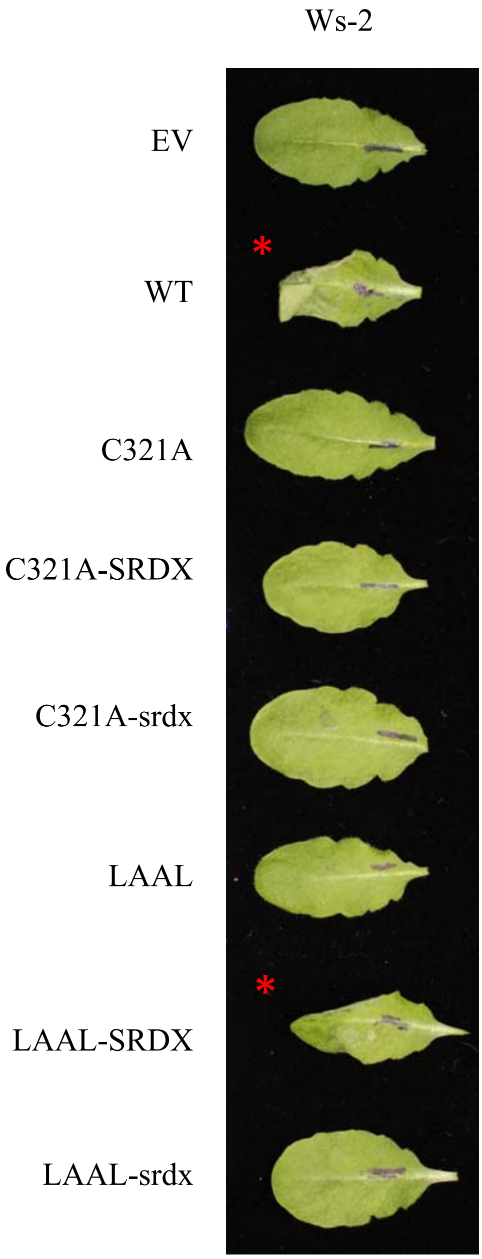

Supplementary Figure 3. The SRDX motif partially restores PopP2^{LAAL} avirulence activity in Arabidopsis. PopP2 variants C-terminally tagged with 6xHA were delivered by *Pseudomonas fluorescens* Pf0-1(T3S) into Ws-2 leaves and photographs were taken 1 day after infiltration. A red asterisk indicates visible cell death. This experiment was conducted 3 times with similar results.

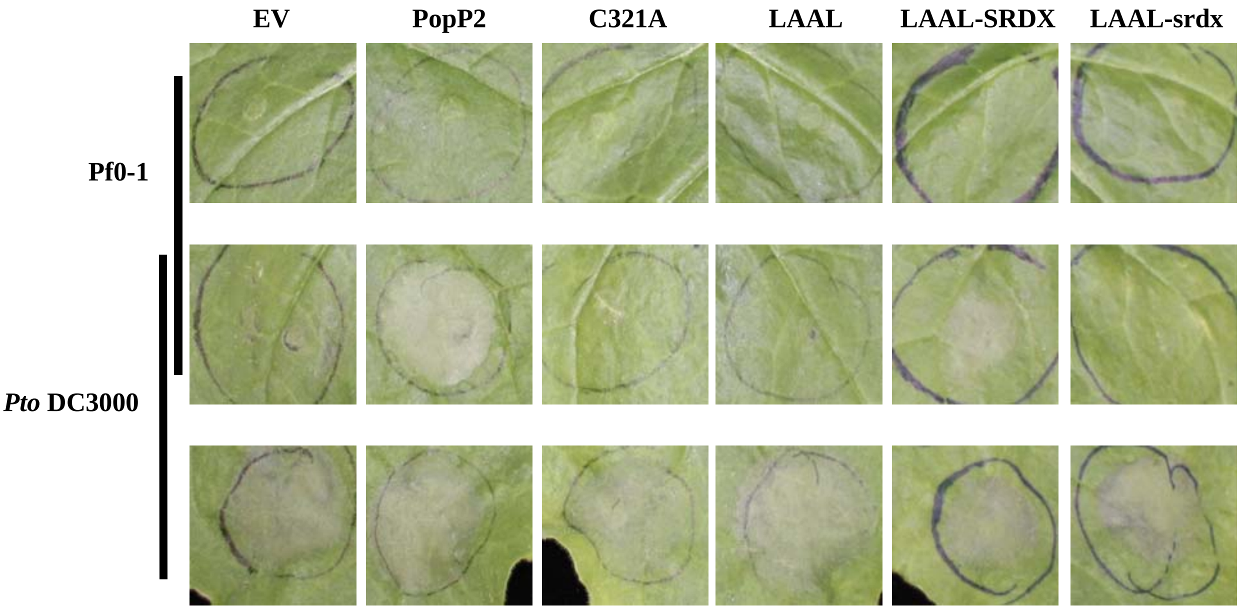

Supplementary Figure 4. The EAR motif is required for PopP2-mediated inhibition of *P. fluorescens* Pf0-1-induced PTI in *N. benthamiana.* $3x10^{8}$ cfu/ml of *P. fluorescens* Pf0-1 carrying empty vector or PopP2 variants (WT, C321A, LAAL, LAAL-SRDX or LAAL-srdx) were infiltrated in 5-week old *N. benthamiana* leaves (in indicated circles as 'Pf0-1'). After 8hours, $2x10^{7}$ cfu/ml of *Pto* DC3000 was infiltrated (in indicated circles as 'DC3000'). *Pto* DC3000-triggered tissue collapse was scored at 48hpi after *Pto* DC3000 infiltration. This experiment was conducted 4 times with similar results.

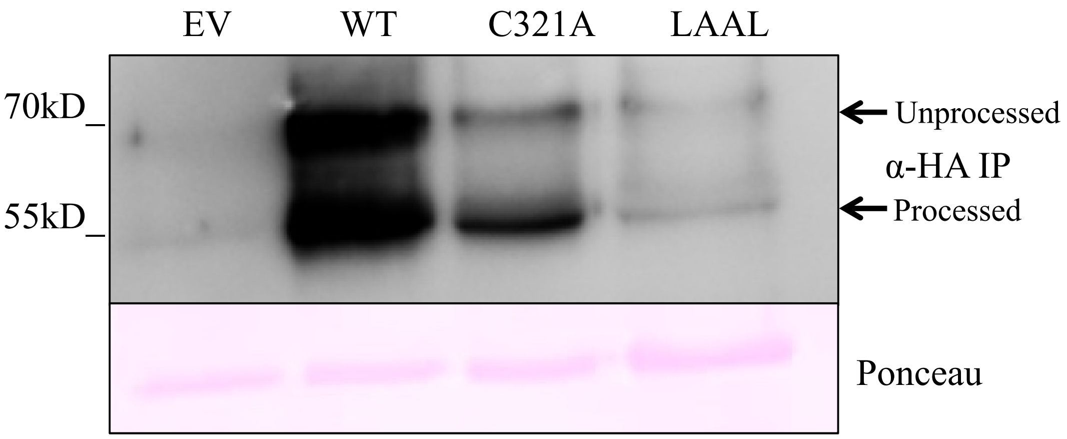

Supplementary Figure 5. *In planta* processing of *Pto* DC3000-delivered AvrRps4N:PopP2:6xHA variants in *N. benthamiana*. The AvrRps4N:PopP2 variants C-terminally tagged with 6xHA were delivered by *Pto* DC3000 ($1x10^9$cfu/ml) in *N. benthamiana*. *N. benthamiana* leaf samples were harvested at 10 hours post infiltration. Protein accumulation of PopP2 variants was confirmed by immunoprecipitation followed by immunoblot analysis using α-HA antibody. Protein loading was confirmed with Ponceau staining.

Corepressor	Chromatin modifier (HDAC)	Repressor recruitment	Cofactor recruitment	References
HOS15	HOS15	Unknown		Zhu et al., 2008
LUG	HDA19	-		Gonzalez et al., 2007
LUH	Unknown	-		Shrestha et al., 2014
SAP18	HDA19	EAR motif		Song & Galbraith, 2006; Hill et al., 2008
SEU	- LUG - HDA19<br>- LUH - Unknown	Unknown	LUFS domain of LUG to form corepressor complex	Sridhar et al., 2004; Sitaraman et al., 2008; Gonzalez et al., 2007; Shrestha et al., 2014
SLK1	- LUG - HDA19<br>- LUH - Unknown	Unknown		Gonzalez et al., 2007<br>Stahle et al., 2009; Shrestha et al., 2014
SLK2	- LUG - HDA19<br>- LUH - Unknown	Unknown		Gonzalez et al., 2007<br>Stahle et al., 2009; Shrestha et al., 2014
TPL	HDA19	EAR motif		Long et al., 2006; Szemenyei et al., 2008; Causier et al., 2012; Krogan et al., 2012
TPR1	HDA19	EAR motif		Zhu et al., 2010; Causier et al., 2012; Lee et al., 2016
TPR2	Unknown, likely HDA19	EAR motif		Causier et al., 2012; Lee et al., 2016
TPR3	Unknown, likely HDA19	EAR motif		Causier et al., 2012; Lee et al., 2016
TPR4	Unknown, likely HDA19	EAR motif		Causier et al., 2012; Lee et al., 2016

Supplementary Table 1. Co-repressor library screened in the Y2H assay. Details of the co-repressor library screened for interaction with PopP2 in the yeast-two-hybrid (Y2H) assay.

Supplementary References

Causier, B., Ashworth, M., Guo, W., and Davies, B. (2012). The TOPLESS interactome: a framework for gene repression in Arabidopsis. *Plant physiology* 158(1)**,** 423-438.

Gonzalez, D., Bowen, A.J., Carroll, T.S., and Conlan, R.S. (2007). The transcription corepressor LEUNIG interacts with the histone deacetylase HDA19 and mediator components MED14 (SWP) and CDK8 (HEN3) to repress transcription. *Molecular and cellular biology* 27(15)**,** 5306-5315.

Hill, K., Wang, H., and Perry, S.E. (2008). A transcriptional repression motif in the MADS factor AGL15 is involved in recruitment of histone deacetylase complex components. *The Plant Journal* 53(1)**,** 172-185.

Krogan, N.T., Hogan, K., and Long, J.A. (2012). APETALA2 negatively regulates multiple floral organ identity genes in Arabidopsis by recruiting the co-repressor TOPLESS and the histone deacetylase HDA19. *Development* 139(22)**,** 4180-4190.

Lee, M.-S., An, J.-H., and Cho, H.-T. (2016). Biological and molecular functions of two EAR motifs of Arabidopsis IAA7. *Journal of Plant Biology* 59(1)**,** 24-32.

Le Roux, C., Huet, G., Jauneau, A., Camborde, L., Tremousaygue, D., Kraut, A., Zhou, B., Levaillant, M., Adachi, H., Yoshioka, H., Raffaele, S., Berthome, R., Coute, Y., Parker, J., Deslandes, L., 2015. A Receptor Pair with an Integrated Decoy Converts Pathogen Disabling of Transcription Factors to Immunity. Cell 161, 1074–1088. doi:10.1016/j.cell.2015.04.025

Long, J.A., Ohno, C., Smith, Z.R., and Meyerowitz, E.M. (2006). TOPLESS regulates apical embryonic fate in Arabidopsis. *Science* 312(5779)**,** 1520-1523.

Shrestha, B., Guragain, B., and Sridhar, V.V. (2014). Involvement of co-repressor LUH and the adapter proteins SLK1 and SLK2 in the regulation of abiotic stress response genes in Arabidopsis. *BMC plant biology* 14(1)**,** 54.

Sitaraman, J., Bui, M., and Liu, Z. (2008). LEUNIG_HOMOLOG and LEUNIG perform partially redundant functions during Arabidopsis embryo and floral development. *Plant physiology* 147(2)**,** 672-681.

Song, C.-P., and Galbraith, D.W. (2006). AtSAP18, an orthologue of human SAP18, is involved in the regulation of salt stress and mediates transcriptional repression in Arabidopsis. *Plant molecular biology* 60(2)**,** 241-257.

Sridhar, V.V., Surendrarao, A., Gonzalez, D., Conlan, R.S., and Liu, Z. (2004). Transcriptional repression of target genes by LEUNIG and SEUSS, two interacting regulatory proteins for Arabidopsis flower development. *Proceedings of the National Academy of Sciences of the United States of America* 101(31)**,** 11494-11499.

Stahle, M.I., Kuehlich, J., Staron, L., von Arnim, A.G., and Golz, J.F. (2009). YABBYs and the transcriptional corepressors LEUNIG and LEUNIG_HOMOLOG maintain leaf polarity and meristem activity in Arabidopsis. *The Plant Cell* 21(10)**,** 3105-3118.

Szemenyei, H., Hannon, M., and Long, J.A. (2008). TOPLESS mediates auxin-dependent transcriptional repression during Arabidopsis embryogenesis. *Science* 319(5868)**,** 1384-1386.

Zhu, J., Jeong, J.C., Zhu, Y., Sokolchik, I., Miyazaki, S., Zhu, J.-K., et al. (2008). Involvement of Arabidopsis HOS15 in histone deacetylation and cold tolerance. *Proceedings of the National Academy of Sciences* 105(12)**,** 4945-

4950.
Zhu, Z., Xu, F., Zhang, Y., Cheng, Y.T., Wiermer, M., Li, X., et al. (2010). Arabidopsis resistance protein SNC1 activates immune responses through association with a transcriptional corepressor. *Proc Natl Acad Sci U S A* 107(31)**,** 13960-13965. doi: 10.1073/pnas.1002828107.
